# A Case-Control Study of Contextual Factors for SARS-CoV-2 Transmission

**DOI:** 10.3389/fpubh.2021.772782

**Published:** 2021-11-04

**Authors:** Andreia Leite, Teresa Leão, Patrícia Soares, Milton Severo, Marta Moniz, Raquel Lucas, Pedro Aguiar, Paula Meireles, Nuno Lunet, Carla Nunes, Henrique Barros

**Affiliations:** ^1^NOVA National School of Public Health, Public Health Research Center, Universidade NOVA de Lisboa, Lisboa, Portugal; ^2^Comprehensive Health Research Center, Universidade NOVA de Lisboa, Lisboa, Portugal; ^3^EPIUnit, Instituto de Saúde Pública da Universidade do Porto, Porto, Portugal; ^4^Departamento de Ciências da Saúde Pública e Forenses, e Educação Médica, Faculdade de Medicina, Universidade do Porto, Porto, Portugal

**Keywords:** SARS-CoV-2, COVID-19, risk factors, transmission, case-control studies

## Abstract

**Background:** Knowledge on the settings and activities associated with a higher risk of SARS-CoV-2 transmission is essential to inform decision-making. We thus designed a case-control study to identify relevant settings for community transmission of severe acute respiratory syndrome coronavirus 2 (SARS-CoV-2) in Portugal.

**Methods:** We evaluated 1,088 cases, identified through the national surveillance system, and 787 community controls, recruited using random digit dialing. Sociodemographic characteristics, individual protective measures, and activities or visited settings were obtained through telephone interview. We report sex-, age-, education-, and citizenship-adjusted odds ratios (aOR) with 95% confidence intervals (95% CI).

**Results:** Household overcrowding (aOR = 1.47; 95% CI 1.14–1.91) and work in senior care (4.99; 1.30–33.08) increased while working remotely decreased the risk of infection (0.30; 0.22–0.42). Going to restaurants/other dining spaces (0.73; 0.59–0.91), grocery stores (0.44; 0.34–0.57) or hair salons (0.51; 0.39–0.66), or the use of public transportation did not present a higher risk of infection (0.98; 0.75–1.29), under existing mitigation strategies. Lower education ( ≤ 4 years vs. tertiary education: 1.79; 1.33–2.42) and no Portuguese citizenship (5.47; 3.43–9.22) were important risk factors.

**Conclusions:** The utilization of public transportation, restaurants, and commercial spaces was not associated with increased risk of infection, under capacity restrictions, physical distancing, use of masks, and hygiene measures. Overcrowding, foreign citizenship, low education and working on-site were positively associated with SARS-CoV-2 infection.

## Introduction

Before extended worldwide vaccination coverage is achieved, non-pharmaceutical measures remain an essential option to control COVID-19 pandemic in most countries (1). Non-pharmaceutical measures range from recommendations to reduce social contacts to nationwide curfews, partial and full lockdowns, and closure of restaurants, bars, and other non-essential services (2). These restrictive measures contribute for a reduction in the incidence of COVID-19 cases but severely affect social and economic activities (3). The identification of the circumstances and the settings that determine a higher risk of severe acute respiratory syndrome coronavirus 2 (SARS-CoV-2) transmission can contribute to inform a more precise implementation of protective measures.

Ecological studies identified individuals' socioeconomic status, and population density, overcrowding, and mobility as relevant infection determinants (4–7). Secondary transmission was shown to occur more frequently among cohabitants than with other contacts, suggesting an increased risk of intrafamilial transmission of SARS-CoV-2 (8). Cohabitants sharing rooms and talking for 30 min or more were at higher risk; among non-cohabitants, the exposure to 1 or more COVID-19 cases, talking for 30 min or more, and the use of ride sharing were associated with a higher risk of infection (9).

While contact (direct or through a vehicle) with an infected person is required for transmission to occur, most individuals cannot identify these contacts, making crucial the identification of settings with higher risk of transmission. In the United States (US) a study of 364 cases identified 27% reported contact with at least one person with known SARS-CoV-2 infection and, of those, more than 50% were in family or work settings (10). From those who did not identify any epidemiological link, 44% had been in gatherings with more than 10 people, 22% had used public transportation, 28% worked in a healthcare setting, and 23% had visited a healthcare setting. Also in the US, a study of 154 cases and 160 controls identified a higher risk of infection among customers of restaurants, bars, or cafes (11). The authors identified that non-Hispanic white, highly educated and those who had 1 or more underlying chronic medical conditions were less often diagnosed with SARS-CoV-2 infection (11). Having gatherings at home (with more or <10 people); going to office settings, gyms, or religious gatherings; or using public transportation did not show significant differences between cases and controls (11).

In France, 66.0% of the infected individuals who were not healthcare professionals identified an epidemiological link; among those, 35.0% of the infections occurred at home (12). From the infections occurring outside the home, 33.1% occurred in the family milieu, 28.8% at work, and 20.8% among friends; sharing meals and offices had a central role in transmission. The risk of infection was positively associated with the number of people living in the household, sharing rides, and going to bars, restaurants, and sports studios, while going to shops, using buses or tramways, and working from home reduced the risk of infection (13). Comparing to public employees, industry workers, drivers, health and social sectors' professionals, and senior executives had a higher risk of infection and those working remotely had lower risk (12). Estimates did not take into account the use of individual protective measures nor the potential confounding of education, nationality, or citizenship (13).

Evidence on the settings with the highest risk of SARS-CoV-2 transmission, taking into account the adherence to individual protection measures, is still scarce. We thus aimed at identifying the settings of transmission of SARS-CoV-2 in a country with extensive use of non-pharmacological interventions by comparing a large sample of SARS-CoV-2 cases and community controls in the largest region of Portugal, Lisbon, and Tagus Valley.

## Materials and Methods

### Design

This case-control study was conducted within the Lisbon and Tagus Valley area, comprising one third of the country's population distributed between urban and rural, high and low population density areas.

Cases were individuals with SARS-CoV-2 infection diagnosed using reverse transcription–polymerase chain reaction and reported to the National System of Epidemiological Surveillance during week 39 of 2020 (September 29-October 4). Controls were identified among residents of the same municipalities as cases but with no self-reported prior SARS-CoV-2 infection diagnosis. Controls were selected using random digit dialing, including landline and mobile phone numbers, with frequency matching for sex, age (within 10-year bands), and municipality. Landline numbers were generated assuming the region prefixes as fixed and randomly generating the remaining digits (4–7); we generated up to 99 numbers for each prefix, totalling 61,040 landline numbers. For mobile phone numbers, we used the main operators' prefixes (91, 93, and 96) and generated the remaining digits (7) using a distribution proportional to that observed among cases and assuming that only 10% of these numbers would belong to a resident in the area and among those, a 20% participation rate. We generated a total of 47,600 random mobile numbers, randomly ordered to avoid preferential contact. We tested landline numbers through call attempts; for mobile numbers, a message was sent mentioning the study and informing of a subsequent contact.

**Figure 1:**
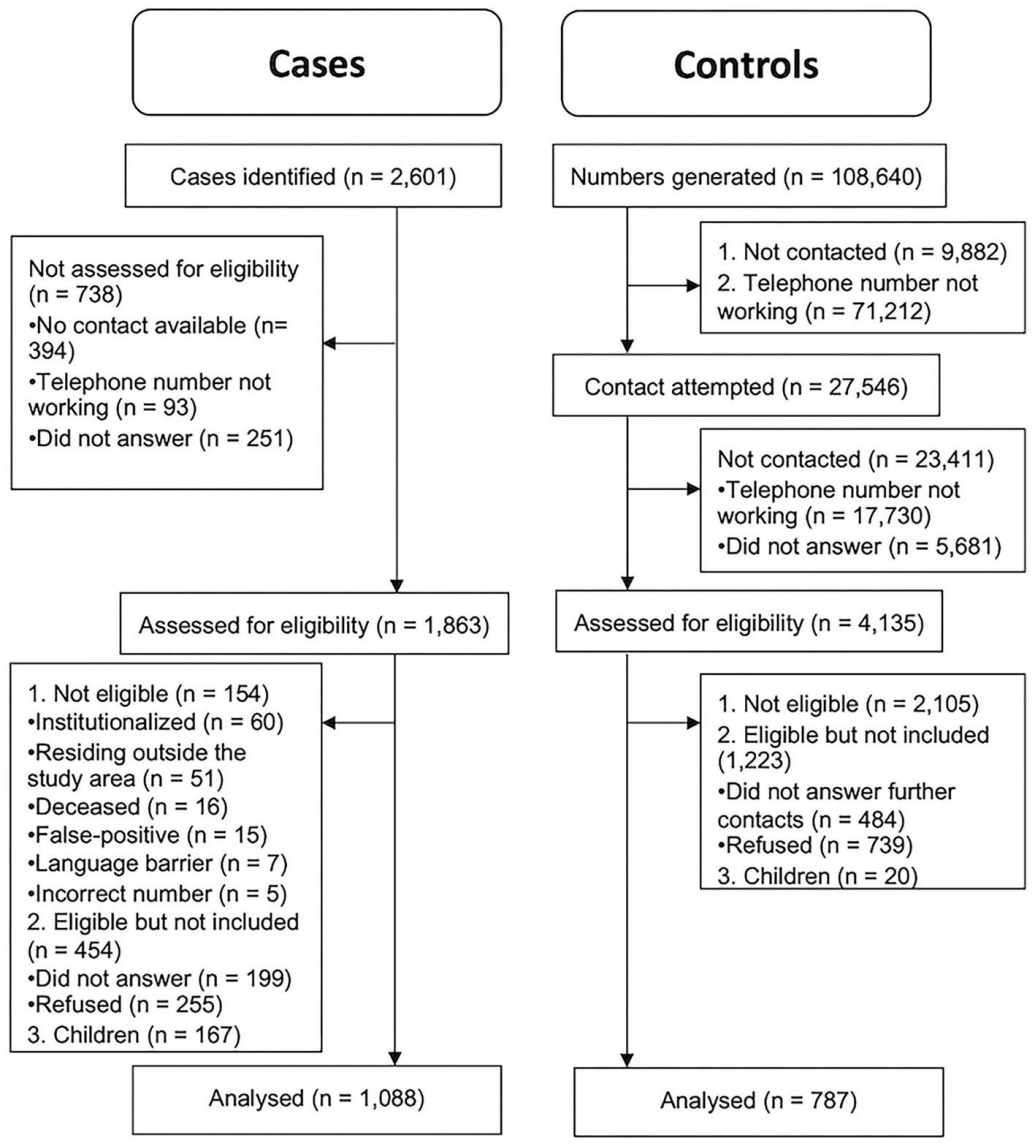
Flow diagram.

We included cases and controls residing in the region during the reference period regardless of their nationality or immigration status. We excluded cases: (1) without a telephone number; (2) institutionalized (e.g., nursing homes, prisons); (3) deceased between the case report and interview dates; and (4) with communication difficulties (language barriers, deafness, serious mental disease, or cognitive impairment) and no proxy available (Figure 1). Telephone numbers from companies or institutions (controls only) were excluded. Additionally, due to the small number of controls aged younger than 18 years successfully recruited (*n* = 20), only adults were considered.

### Data Collection

We developed a questionnaire to collect information on social, demographic, and behavioral factors potentially related to SARS-CoV-2 infection. Data were collected between October 2 and November 6, 2020, through a 20-min computer-assisted telephone interview. Proxies (*n* = 14) were used when eligible participants were unable to answer due to cognitive impairment, deafness, or language barriers. The final sample size included 1,088 cases and 787 controls.

### Definition of Variables

We included as dependent variables those related to contextual factors that could increase the risk of transmission of SARS-CoV-2. To assess housing conditions, we analyzed the number of individuals per room, i.e., the number of individuals in the household divided by the number of rooms with area of 4 m^2^ or more, excluding storage rooms, vestibules, and bathrooms (14). Occupation and professional status can be linked to a higher risk of transmission (13), as such, individuals were classified as unemployed, students, retired, otherwise inactive, or employed. The latter were then classified into economic activity sectors (construction, cleaning, education, healthcare, industry, restaurants, senior care, and other). Because remote work has been implemented as a measure to reduce the risk of infection, we compared those who did and did not work remotely at least 1 day during the reference period; this analysis was restricted to employed and unemployed individuals.

The use of public transportation (tube, bus, train, boat, company transport, and others), restaurants and other dining spaces (coffee shops, bakeries, pastry shops), grocery stores (including supermarkets), other commercial spaces (including shopping malls), hair salons (including aesthetic centres), and gyms (including sports studios) was assessed. History of use was considered present only for individuals staying for at least 15 min during the reference period.

Adherence to individual protection measures was also assessed, including frequency of use of face masks, face shields, and gloves (always or almost always, sometimes, never or almost never) and hand hygiene (<3 times, 3–5 times, six or more times a day, to help participants in capturing their perception of daily frequency of hands hygiene).

Sex, age, municipality, citizenship (with or without Portuguese citizenship), and education level according to the highest level attained (up to 4 years, 6 years, 9 years, 12 years, and tertiary education) were deemed potential confounders.

Furthermore, we collected data regarding previous known contacts with persons who were diagnosed with SARS-CoV-2 infection during the reference period.

### Reference Period

For cases, questions referred to the 14 days prior to symptom onset or, in case of asymptomatic individuals, to the 14 days before the date of the first positive sample collection. For controls, the reference period was defined as the 14 days prior to the interview. During field work, further control measures were implemented, starting October 15 (15). These included rules regarding stores' opening times, remote work whenever possible, and limitation of the number of individuals in events, among others. To ensure comparability and avoid bias, all controls interviewed after October 15 had the first 2 weeks of October as the reference period for exposures. As such, the date used to determine the reference period for cases was situated between September 1 and October 4 and for controls between October 2 and October 14.

### Analysis

We fitted unconditional logistic regression models to estimate crude and adjusted odds ratios (aOR) and corresponding 95% CIs (95% Confidence Intervals). Analyses were adjusted for sex, age, education level, and citizenship status. We conducted a sensitivity analysis including only individuals without a known contact with SARS-CoV-2 infection cases (10, 11). Adherence to individual protection measures was also described but, due to the high levels of adherence and balance in cases and controls, was not included in the regression models.

Given the low percentage (<1.5%) of missing data (Table 1), a complete case analysis with pairwise deletion was conducted (16, 17). Data were analyzed using R version 4.0.3 (18).

**Table 1:** Sample characteristics according to sociodemographic aspects, contacts, and spaces among cases of SARS-CoV-2 and controls from the Lisbon and Tagus Valley Region in September and October 2020.

	**No. (%)**	
	**Cases**	**Controls**
	**(*n* = 1,088)**	**(*n* = 787)**
**Sex**		
Female	637 (58.5)	490 (62.3)
Male	450 (41.4)	297 (37.7)
Missing	1 (0.1)	0 (0.0)
**Age, years**		
Mean (SD)	44.3 (15.7)	52.9 (16.0)
Median [min-max]	43.0 [18.0–94.0]	52.0 [18.0–99.0]
**Education level**		
≤ 4 years	145 (13.3)	96 (12.2)
6 years	94 (8.6)	29 (3.7)
9 years	169 (15.5)	93 (11.8)
12 years	363 (33.4)	193 (24.5)
Tertiary	316 (29.0)	375 (47.6)
Missing	1 (0.1)	1 (0.1)
**Citizenship**		
Portuguese	943 (86.7)	760 (96.6)
Non-portuguese	129 (11.9)	19 (2.4)
Missing	16 (1.5)	8 (1.0)
**Contact with known case**		
No	623 (57.3)	763 (97.0)
Yes	465 (42.7)	23 (2.9)
Missing	0 (0)	1 (0.1)
**No. of people per room (area** **≥4 m**^**2**^**)**		
<1	775 (71.2)	662 (84.1)
≥1	311 (28.6)	119 (15.1)
Missing	2 (0.2)	6 (0.8)
**Occupation/professional situation**		
Construction	36 (3.3)	7 (0.9)
Cleaning	43 (4.0)	13 (1.7)
Education	44 (4.0)	34 (4.3)
Healthcare	86 (7.9)	31 (3.9)
Industry	22 (2.0)	7 (0.9)
Restaurant industry	39 (3.6)	12 (1.5)
Senior care	33 (3.0)	2 (0.3)
Other occupation	458 (42.1)	393 (49.9)
Student	57 (5.2)	23 (2.9)
Retired	117 (10.8)	198 (25.2)
Unemployed	99 (9.1)	45 (5.7)
Another professional situation	47 (4.3)	21 (2.7)
Missing	7 (0.6)	1 (0.1)
Remote working^*^		
Unemployed	99 (9.1)	46 (5.8)
No	693 (63.7)	338 (42.9)
Yes	73 (6.7)	159 (20.2)
Missing	223 (20.5)	244 (31.0)
**Used public transportation**		
No	854 (78.5)	661 (84.0)
At least once	231 (21.2)	125 (15.9)
Missing	3 (0.3)	1 (0.1)
**Went to restaurants and other dining spaces**		
No	571 (52.5)	327 (41.6)
At least once	513 (47.2)	455 (57.8)
Missing	4 (0.4)	5 (0.6)
**Went to grocery stores**		
No	326 (30.0)	127 (16.1)
At least once	755 (69.4)	659 (83.7)
Missing	7 (0.6)	1 (0.1)
**Went to shopping malls/shops**		
No	825 (75.8)	503 (63.9)
At least once	258 (23.7)	283 (36.0)
Missing	5 (0.5)	1 (0.1)
**Went to hair salons/aesthetic centers**		
No	927 (85.2)	589 (74.8)
At least once	156 (14.3)	197 (25.0)
Missing	5 (0.5)	1 (0.1)
**Went to gyms/sports studios**		
No	1003 (92.2)	723 (91.9)
At least once	81 (7.5)	61 (7.7)
Missing	4 (0.4)	3 (0.4)
**Face masks in public spaces or workplace**		
Always or almost always	1041 (95.7)	757 (96.2)
Sometimes	30 (2.8)	19 (2.4)
Never or almost never	14 (1.3)	7 (0.9)
Missing	3 (0.3)	4 (0.5)
**Face shields in public spaces**		
Always or almost always	33 (3.0)	11 (1.4)
Sometimes	23 (2.1)	13 (1.7)
Never or almost never	1029 (94.6)	759 (96.4)
Missing	3 (0.3)	4 (0.5)
**Gloves**		
Always or almost always	79 (7.3)	30 (3.8)
Sometimes	102 (9.4)	47 (6.0)
Never or almost never	904 (83.1)	706 (89.7)
Missing	3 (0.3)	4 (0.5)
**Hands' hygiene (times/day)**		
≥6 times	887 (81.5)	647 (82.2)
3–5 times	176 (16.2)	125 (15.9)
<3 times	21 (1.9)	11 (1.4)
Missing	4 (0.4)	4 (0.5)

* *Restricted to those employed and unemployed, remaining observations deemed as missing values*.

### Ethical and Data Protection Issues

This study was approved by the ethics committees from the institutions involved. Verbal informed consent was obtained from the participants prior to the interview. Guidelines from data protection officers from the institutions involved were also followed.

## Results

The characteristics of the 1,088 cases and 787 controls are presented in Table 1. Fifty-eight percent of cases and 62.3% of controls were female. Cases had a mean (SD) age of 44.3 (15.7) years, while controls had a mean (SD) age of 52.9 (16.0) years. Regarding the use of facemasks, 95.7% of cases and 96.2% of controls reported to use them always or almost always. Cases and controls also reported high frequency of hand hygiene (81.5% of cases and 82.2% of controls reported to wash hands/use hand sanitiser six or more times a day).

The risk of infection was 2 to 4 times higher among individuals with lower education level compared with those with tertiary education (Table 2). Participants without Portuguese citizenship had a relative risk of infection of 5.47 (95% CI, 3.43–9.22). Unsurprisingly, the risk of infection among those with previous known contact with a SARS-CoV-2 infection case was particularly high (aOR, 24.76; 95% CI, 16.45–39.18).

**Table 2:** Risk of infection of SARS-CoV-2 among cases and controls from the Lisbon and Tagus Valley Region in September and October 2020 according to education level, citizenship status, and known contact with a case.

	**Odds ratio (95% CI)**	***P*-value**
**Education level**		
Reference: Tertiary	(*n* = 1,873)	
≤ 4 years	1.79 (1.33–2.42)	<0.001
6 years	3.85 (2.50–6.08)	<0.001
9 years	2.16 (1.61–2.90)	<0.001
12 years	2.23 (1.78–2.81)	<0.001
**Citizenship**		
Reference: Portuguese	(*N* = 1,851)	
Non-portuguese	5.47 (3.43–9.22)	<0.001
**Known contact with a case**		
Reference: No	(*N* = 1,874)	
Yes	24.76 (16.45–39.18)	<0.001

*95% CI, 95% Confidence Interval*.

The results of the main and sensitivity analyses, including and excluding participants with a known contact with a case, are presented in Tables 3, 4, respectively. An increased risk of infection was identified among those living in a house with 1 or more individuals per room (aOR, 1.47; 95% CI, 1.14–1.91) and for individuals working in senior care (aOR, 4.99; 95% CI, 1.30–33.08). A lower risk of infection was identified among those who reported working remotely compared with those working in-office (aOR, 0.30; 95% CI, 0.22–0.42) and among individuals who went to restaurants and other dining spaces (aOR, 0.73; 95% CI, 0.59–0.91), grocery stores (aOR, 0.44; 95% CI, 0.34–0.57), shopping malls/shops (aOR, 0.51; 95% CI, 0.40–0.64), or hair salons/aesthetic centres (aOR, 0.51; 95% CI, 0.39–0.66) at least once during the reference period. We did not find any association with the use of public transportation at least once (aOR, 0.98; 95% CI, 0.75–1.29) or going to the gym at least once (aOR, 1.04; 95% CI, 0.71–1.55).

**Table 3:** Risk of infection of SARS-CoV-2 among cases and controls from the Lisbon and Tagus Valley Region in September and October 2020.

**Variable**	**Crude odds ratio (95% CI)**	***P*-value**	**Adjusted odds ratio (95% CI)^**a**^**	***P*-value**
**No. of people per room (area** **≥** **4 m**^**2**^**)**				
Reference: <1	(*n* = 1,867)		(*n* = 1,840)	
≥1	2.23 (1.77–2.83)	<0.001	1.47 (1.14–1.91)	0.004
**Occupation/professional situation**				
Reference: Education	(*n* = 1,867)		(*n* = 1,840)	
Construction	3.97 (1.65–10.73)	0.003	1.11 (0.42–3.17)	0.842
Cleaning	2.56 (1.21–5.64)	0.016	0.89 (0.39–2.08)	0.782
Restaurant industry	2.51 (1.17–5.68)	0.022	0.93 (0.41–2.19)	0.860
Healthcare	2.14 (1.17–3.96)	0.014	1.87 (0.98–3.58)	0.058
Industry	2.43 (0.96–6.75)	0.070	0.88 (0.33–2.58)	0.808
Senior care	12.75 (3.53–82.08)	<0.001	4.99 (1.30–33.08)	0.041
Other occupation	0.90 (0.56–1.43)	0.660	0.66 (0.40–1.09)	0.109
Student	1.92 (1.00–3.74)	0.053	0.44 (0.21–0.93)	0.030
Retired	0.46 (0.27–0.75)	0.002	0.54 (0.29–1.00)	0.049
Unemployed	1.70 (0.96–3.01)	0.068	0.80 (0.43–1.49)	0.484
Another professional situation	1.73 (0.88–3.45)	0.115	0.91 (0.43–1.95)	0.815
**Remote working**				
Reference: No (working in-office)	(*n* = 1,408)		(*n* = 1,386)	
Unemployed	1.05 (0.73–1.54)	0.799	0.75 (0.50–1.14)	0.170
Yes	0.22 (0.16–0.30)	<0.001	0.30 (0.22–0.42)	<0.001
**Used public transportation**				
Reference: No	(*n* = 1,871)		(*n* = 1,844)	
At least once	1.43 (1.13–1.82)	0.003	0.98 (0.75–1.29)	0.897
**Went to restaurants and other dining spaces**				
Reference: No	(*n* = 1,866)		(*n* = 1,839)	
At least once	0.65 (0.54–0.78)	<0.001	0.73 (0.59–0.91)	0.005
**Went to grocery stores**				
Reference: No	(*n* = 1,867)		(*n* = 1,840)	
At least once	0.45 (0.35–0.56)	<0.001	0.44 (0.34–0.57)	<0.001
**Went to shopping malls/shops**				
Reference: No	(*n* = 1,869)		(*n* = 1,842)	
At least once	0.56 (0.45–0.68)	<0.001	0.51 (0.40–0.64)	<0.001
**Went to hair salons/aesthetic centers**				
Reference: No	(*n* = 1,869)		(*n* = 1,842)	
At least once	0.50 (0.40–0.64)	<0.001	0.51 (0.39–0.66)	<0.001
**Went to gyms/sports studios**				
Reference: No	(*n* = 1,868)		(*n* = 1,841)	
At least once	0.96 (0.68–1.36)	0.804	1.04 (0.71–1.55)	0.832

a *Adjusted for sex, age, education level, and citizenship. 95% CI, 95% Confidence Interval*.

**Table 4:** Risk of infection of SARS-CoV-2 among cases and controls from the Lisbon and Tagus Valley Region in September and October 2020.

**Variable**	**Crude odds ratio (95% CI)**	***P*-value**	**Adjusted odds ratio (95% CI)^**a**^**	***P*-value**
**Number of people per room (area** **≥** **4 m**^**2**^**)**				
Reference: <1	(*n* = 1,378)		(*n* = 1,360)	
≥1	2.09 (1.60–2.73)	<0.001	1.47 (1.10–1.97)	0.010
**Occupation/professional situation**				
Reference: Education	(*n* = 1,380)		(*n* = 1,362)	
Civil construction	3.75 (1.42–10.90)	0.010	1.01 (0.34–3.20)	0.986
Cleaning	2.88 (1.26–6.86)	0.014	1.12 (0.46–2.84)	0.807
Restaurant industry	2.29 (0.96–5.68)	0.066	0.79 (0.31–2.09)	0.631
Healthcare	2.05 (1.01–4.23)	0.048	1.74 (0.81–3.77)	0.155
Industry	2.14 (0.74–6.56)	0.165	0.76 (0.24–2.53)	0.651
Senior care	10.62 (2.69–71.27)	0.003	4.03 (0.94–28.25)	0.093
Other occupation	0.87 (0.50–1.54)	0.628	0.62 (0.34–1.15)	0.125
Student	1.85 (0.87–3.96)	0.110	0.41 (0.18–0.97)	0.042
Retired	0.43 (0.24–0.79)	0.006	0.49 (0.24–1.01)	0.052
Unemployed	1.69 (0.87–3.30)	0.124	0.80 (0.39–1.66)	0.543
Another professional situation	1.31 (0.59–1.54)	0.511	0.65 (0.27–1.58)	0.343
**Remote working**				
Reference: No (working in-office)	(*n* = 1,016)		(*n* = 1,003)	
Unemployed	1.09 (0.72–1.67)	0.680	0.80 (0.51–1.28)	0.353
Yes	0.21 (0.14–0.30)	<0.001	0.27 (0.18–0.40)	<0.001
**Used public transportation**				
Reference: No	(*n* = 1,384)		(*n* = 1,366)	
At least once	1.73 (1.32–2.25)	<0.001	1.16 (0.86–1.57)	0.333
**Went to restaurants and other dining spaces**				
Reference: No	(*n* = 1,378)		(*n* = 1,360)	
At least once	0.71 (0.57–0.88)	0.002	0.79 (0.61–1.02)	0.069
**Went to grocery store**s				
Reference: No	(*n* = 1,382)		(*n* = 1,364)	
At least once	0.48 (0.37–0.62)	<0.001	0.47 (0.35–0.63)	<0.001
**Went to shopping malls/shops**				
Reference: No	(*n* = 1,383)		(*n* = 1,365)	
At least once	0.61 (0.48–0.77)	<0.001	0.56 (0.43–0.73)	<0.001
**Went to hair salons/aesthetic centers**				
Reference: No	(*n* = 1,383)		(*n* = 1,365)	
At least once	0.54 (0.41–0.71)	<0.001	0.58 (0.43–0.78)	<0.001
**Went to gyms/sports studios**				
Reference: No	(*n* = 1,379)		(*n* = 1,361)	
At least once	1.13 (0.77–1.67)	0.528	1.23 (0.79–1.92)	0.359

*Analysis restricted to the cases and controls reporting no known contact with cases of SARS-CoV-2 infection in the reference period*.

a *Adjusted for sex, age, education level, and citizenship. 95% CI, 95% Confidence Interval*.

The results for the analysis restricted to those without known contact with a SARS-CoV-2 infection case remained essentially unchanged (Table 4).

## Discussion

We identified several settings and activities associated with SARS-CoV-2 infection, including socioeconomic, work, and household-related conditions. Cases more frequently had lower education, no Portuguese citizenship, and lived in crowded households. The use of public transportation, dining places, or other commercial areas was not associated with a higher risk of infection, after adjusting for sex, age, citizenship, and education. We identified a strong protective effect of working remotely.

Our results emphasize the role of social determinants of health in the transmission of infection, as suggested by others (7, 19). Individuals with lower education tend to work in manual jobs, have lower income, and are subject to worse working and housing conditions. They might also present a poorer health literacy. Migrants (defined here as individuals without Portuguese citizenship) may also experience less favorable living and working conditions, namely overcrowding (20), as observed by Hayward et al. (21) in their systematic review about the risk factors for COVID-19 among migrants in high income-countries. Overcrowding increases proximity to others, which results in increased transmission risk from undiagnosed COVID-19 cases but also in important limitations to isolation measures after diagnosis.

Remote work was protective, reducing by 69% the odds of SARS-CoV-2 infection. Galmiche et al. (13) also identified a protective effect of remote work, though of smaller magnitude. There might be some residual confounding as those able to work remotely tend to have better living conditions, and better comply with individual protective measures. On the contrary, by classifying all professionals with at least a day working remotely as remote workers, we might have even underestimated the effect of such a measure.

We identified other work-related risks. In particular, working in senior care as in healthcare was a risk factor for infection. This finding was also reported by Galmiche et al. (12) and might be explained by the higher risk of infection in these settings but also by the higher frequency of testing among those professionals compared with the remaining population.

There was a crude association of public transportation with risk of infection, but it was largely attenuated after adjustment for sex, age, education, and citizenship. These results are in line with a previous ecological study in the same geographical setting, which underlined the role of socioeconomic aspects rather than the use of public transportation (22). Similarly, dining areas or other commercial spaces did not increase the risk of infection, even after adjustment for education or citizenship. While most of our results are in agreement with those from Fisher et al. (11) and Galmiche et al. (13), that is not the case for restaurants and other dining areas. Those studies reported an increased risk among those who dined in a restaurant or were in a bar. Discrepant results might be explained by contextual differences, especially considering the collective and individual protective measures undertaken in each country. First, in Portugal, bars remained closed since the beginning of the pandemic. Second, early on in the pandemic, the government required strict measures, such as the use of masks, physical distancing, environmental hygiene guidelines and limits in the number of persons for public indoor spaces in Portugal. Our results show that most respondents used masks “always and almost always,” which show a high compliance to the individual preventive measures. Another explanation may be residual confounding, as going to restaurants or shops could be a proxy for wealth, which, as abovementioned, has a protective effect against infection.

As such, this study shows that, if incidence rates of SARS-CoV-2 infections, hospitalizations and deaths due to COVID-19 rise, two actions must be taken to limit the control of its transmission. First, it is important to know the population groups where the transmission is occurring and the underlying conditions that may be facilitating it. If needed, the living and working conditions of those in most vulnerable circumstances must be improved, as suggested by the International Labour Organization (23) and the ECDC (24). Second, remote work, physical distancing and mask use in public places as restaurants and bars, commercial spaces and public transports, and intensification of ventilation measures and surfaces' hygiene, must be implemented, as they may contribute for the reduction of the risk of transmission in public settings, while sparing the economic and social side effects of lockdowns. However, it must be stressed that the populations' compliance to distancing and stay-at-home measures tends to decrease through time (25, 26) and, thus, the reintroduction of these measures must be complemented with sensitizing messages and enforcement efforts.

Study limitations need to be considered. The procedure for recruitment of controls might have resulted in selection bias. Controls presented a higher median age, probably due to the fact that older individuals were more prone to answer telephone calls and available to participate in the study during working hours. However, contacts were tried at different hours and during week-ends. The proportion of tertiary education among controls was higher than previously described for the region, and controls consisted of a lower proportion of foreign-born individuals (27, 28). It should be noted that controls were identified from the municipalities with higher incidence of SARS-CoV-2, which are urban municipalities with populations with higher education levels. Nevertheless, these differences might be due to the fact that highly educated individuals were probably more aware of the importance of research thus more willing to participate.

Second, there are potential information biases, including social desirability and recall bias. The former might have occurred in the reporting of preventive behaviors during the reference period, and an inaccurate description of the exposure conditions. This is particularly important for cases who might have overestimated their adherence to preventive behaviors, thus weakening or reversing true associations. This source of bias might have been particularly relevant when reporting the use of face masks or hand hygiene habits, as indicated by high levels of adherence in both cases and controls. Recall bias could have happened, particularly in the later stages of field work, as cases had an average 26 days between the midpoint of the reference period and data collection, while for controls the difference was 16 days. Furthermore, controls might have experienced asymptomatic infection, for which they were not tested, thus being incorrectly assigned as controls instead of cases.

Third, knowledge of the dynamics of transmission is still limited, thus challenging the control of confounding factors. Age, sex, and municipality were considered at the design stage, with age and sex also included in the analysis to avoid residual confounding. Furthermore, we included citizenship and education as confounders. We did not formally consider time in our analysis, as there was a small difference in date used to define the reference period of cases and controls, measures implemented at the national level did not change between these periods.

Fourth, transmission dynamics vary in time and place, according to population characteristics, epidemic activity, measures implemented to control the pandemic, and normalization of the use or non-use of protective behaviors. These aspects should be considered when attempting to generalize these results.

Despite these limitations, our study has several strengths. First, the study design allowed us to obtain estimates of the relative risk as we used incident cases and community controls (29). Second, we obtained all cases through the nationwide official surveillance system, which, by law, demands clinical and laboratory notification of cases, reducing the risk of bias in case selection. Third, we collected detailed information regarding the characteristics of the individuals and the public settings visited by participants during the reference period. Fourth, the sample size allowed comprehensive and robust identification of SARS-CoV-2 individual factors and settings of its transmission.

## Conclusions

Data strongly supports that lower socioeconomic status, including citizenship, and overcrowding increase the risk of infection, while remote working protects against it. Use of public transportation, dining options, and commercial spaces under the implementation of capacity restrictions, physical distancing, use of masks, and surface hygiene is not associated with an increased risk of infection. While public settings did not significantly contribute to the transmission of SARS-CoV-2, work and home were the settings in which infection occurs most frequently, especially among individuals from lower socioeconomic backgrounds. These findings can guide application of fine-tuned non-pharmaceutical measures while vaccine access remains limited.

## Data Availability Statement

Data will be shared with investigator support, after approval of a proposal, with a signed data access agreement and the final manuscript needs to be approved by the institutional Scientific Committee of the Institute of Public Health, University of Porto and NOVA-National School of Public Health.

## Ethics Statement

This study was approved by the Ethics Committees from the institutions involved (Regional Administration of Health of Lisbon and Tagus Valley, NOVA National School of Public Health and Institute of Public Health, Univeristy of Porto). Verbal informed consent was obtained from the participants prior to the interview.

## Author Contributions

HB and CN conceptualized the study. MS and PS performed data analysis. AL and TL supervised data collection and wrote the first draft of the manuscript. All authors commented on previous versions of the manuscript, contributed to study design, interpretation of results, revision, read and approved the final manuscript.

## Funding

This study has been partially funded by the Regional Administration of Health of Lisbon and Tagus Valley, the NOVA National School of Public Health, and National Funds through Foundation for Science and Technology, I.P., within the scope of the Epidemiology Research Unit–Institute of Public Health, University of Porto (EPIUnit) [UIDB/04750/2020].

## Conflict of Interest

The authors declare that the research was conducted in the absence of any commercial or financial relationships that could be construed as a potential conflict of interest. This study has been partially funded by the Regional Administration of Health of Lisbon and Tagus Valley, but the entity did not interfere with the study design, results interpretation or policy implications.

## Publisher's Note

All claims expressed in this article are solely those of the authors and do not necessarily represent those of their affiliated organizations, or those of the publisher, the editors and the reviewers. Any product that may be evaluated in this article, or claim that may be made by its manufacturer, is not guaranteed or endorsed by the publisher.

## References

[1] European Centre for Disease Prevention and Control. Data on Country Response Measures to COVID-19 [Internet]. (2021). Available online at: https://www.ecdc.europa.eu/en/publications-data/download-data-response-measures-covid-19 (accessed June 20, 2021).

[2] European Centre for Disease Prevention and Control. Considerations relating to social distancing measures in response to the COVID-19 epidemic. Stockholm: European Centre for Disease Prevention and Control (2020).

[3] Deb P Furceri D Ostry J Tawk N. The economic effects of COVID-19 containment measures. IMF Work Pap. (2020) 20:44. 10.5089/9781513550251.001

[4] Kermarck W McKendrick A. Contributions to the mathematical theory of epidemics. Bull Math Biol. (1991) 53:33–55. 10.1016/S0092-8240(05)80040-0 2059741

[5] Kraemer MUG Yang CH Gutierrez B Wu CH Klein B Pigott DM. The effect of human mobility and control measures on the COVID-19 epidemic in China. Science. (2020) 497:493–7. 10.1126/science.abb4218 32213647 PMC7146642

[6] Li R Richmond P Roehner BM. Effect of population density on epidemics. Phys A Stat Mech Appl. (2018) 510:713–24. 10.1016/j.physa.2018.07.025

[7] Bambra C Riordan R Ford J Matthews F. The COVID-19 pandemic and health inequalities. J Epidemiol Community Health. (2020) 74:964–8. 10.1136/jech-2020-214401 32535550 PMC7298201

[8] Lei H Xu X Xiao S Wu X Shu Y. Household transmission of COVID-19-a systematic review and meta-analysis. J Infect. (2020) 81: 979–97. 10.1016/j.jinf.2020.08.033 32858069 PMC7446647

[9] Ng OT Marimuthu K Koh V Pang J Linn KZ Sun J. SARS-CoV-2 seroprevalence and transmission risk factors among high-risk close contacts: a retrospective cohort study. Lancet Infect Dis. (2020) 21:333–43. 10.1016/S1473-3099(20)30833-1 33152271 PMC7831879

[10] Marshall K Vahey GM McDonald E Tate JE Herlihy R Midgley CM. Exposures before issuance of stay-at-home orders among persons with laboratory-confirmed COVID-19 - Colorado, March (2020). MMWR Morb Mortal Wkly Rep. (2020) 69:847–9. 10.15585/mmwr.mm6926e4 32614809 PMC7332095

[11] Fisher KA Tenforde MW Feldstein LR Lindsell CJ Shapiro NI Files DC. Community and close contact exposures associated with COVID-19 among symptomatic adults ≥18 years in 11 outpatient health care facilities — United States, July (2020). MMWR Morb Mortal Wkly Rep. (2020) 69:1258–64. 10.15585/mmwr.mm6936a5 32915165 PMC7499837

[12] Galmiche S Charmet T Schaeffer L Paireau J Grant R Cheny O. Etude des facteurs sociodémographiques, comportements et pratiques associés à l'infection par le SARS-CoV-2 (ComCor). Institut Pasteur Paris (2020).

[13] Galmiche S Charmet T Schaeffer L Paireau J Grant R Chény O. Exposures associated with SARS-CoV-2 infection in France: a nationwide online case-control study. Lancet Reg Heal Eur. (2021) 7:100148. 10.1016/j.lanepe.2021.100148 34124709 PMC8183123

[14] Eurostat. Methodological Guidelines and Description of EU-SILC Target Variables. Luxembourg: Eurostat (2018). p. 167–81.

[15] Conselho de Ministros. Resolução do Conselho de Ministros 88/2020. Diário da República - I Série-B. Lisbon: Conselho de Ministros (2020). p. 35-(2)−35-(15).

[16] Bartlett JW Harel O Carpenter JR. Asymptotically unbiased estimation of exposure odds ratios in complete records logistic regression. Am J Epidemiol. (2015) 182:730–6. 10.1093/aje/kwv114 26429998 PMC4597800

[17] Pigott TD. A review of methods for missing data. Educ Res Eval. (2001) 7:353–83. 10.1076/edre.7.4.353.8937

[18] R Core Team. R: A Language and Environment for Statistical Computing. Vienna: R Foundation for Statistical Computing (2019).

[19] Horton R. Offline: COVID-19 is not a pandemic. Lancet. (2020) 396:874. 10.1016/S0140-6736(20)32000-6 32979964 PMC7515561

[20] Eurostat. Migrant Integration Statistics–Housing [Internet]. Eurostat Statistics Explained. (2021). Available online at: https://ec.europa.eu/eurostat/statistics-explained/index.php?title=Migrant_integration_statistics_-_housing (accessed September 29, 2021).

[21] Hayward SE Deal A Cheng C Crawshaw A Orcutt M Vandrevala TF. Clinical outcomes and risk factors for COVID-19 among migrant populations in high-income countries: a systematic review. J Migr Heal. (2021) 3:1–19. 10.1016/j.jmh.2021.100041 33903857 PMC8061095

[22] Severo M Ribeiro AI Lucas R Leão T Barros H. Urban rail transportation and SARS-Cov-2 infections: an ecological study in the Lisbon metropolitan area. Front Public Heal. (2021) 9:611565. 10.3389/fpubh.2021.611565 33614581 PMC7887317

[23] International Labour Organization. R115–Workers' Housing Recommendation [Internet]. (2021). Available online at: https://www.ilo.org/dyn/normlex/en/f?p=NORMLEXPUB:12100:0::NO::P12100_ILO_CODE:R115 (accessed September 29, 2021).

[24] European Centre for Disease Prevention Control. Reducing COVID 19 Transmission Strengthening Vaccine Uptake Among Migrant Populations in the EU/EEA. Stockholm: European Centre for Disease Prevention Control (2021).

[25] Petherick A Goldszmidt R Andrade EB Furst R Hale T Pott A. A worldwide assessment of changes in adherence to COVID-19 protective behaviours and hypothesized pandemic fatigue. Nat Hum Behav. (2021) 5:1145–60. 10.1038/s41562-021-01181-x 34345009

[26] Goldstein P Yeyati EL Sartorio L. Lockdown fatigue: the diminishing effects of quarantines on the spread of COVID-19. Covid Econ. (2021) 67, 1–23. 10.21203/rs.3.rs-621368/v1

[27] Administração Regional de Saúde de Lisboa e Vale do Tejo. Perfil Regional Saude LVT. Lisboa: Administração Regional de Saúde de Lisboa e Vale do Tejo (2017)

[28] Santos AP Leite AH. Plano Local de Saúde 2014-2016. Public Health Unit. Amadora (2014).

[29] Rodrigues L Kirkwood BR. Case-control designs in the study of common diseases: updates on the demise of the rare disease assumption and the choice of sampling scheme for controls. Int J Epidemiol. (1990) 19:205–13. 10.1093/ije/19.1.205 2190942

